# Efficacy of intramedullary bridge fixation for midshaft clavicle fractures: a retrospective analysis of a novel technique

**DOI:** 10.1186/s10195-024-00771-5

**Published:** 2024-06-12

**Authors:** Tianyong Ma, Huan Su, Yihong Lu, Junping Chen, Weiyuan Tan, Fang Lei, Dewei Wang

**Affiliations:** Second Department of Orthopedics, Fifth Affiliated Hospital of Zunyi Medical University, No. 1439, Zhufeng Avenue, Doumen District, Zhuhai, 519100 China

**Keywords:** Midshaft clavicle fracture, Ortho-bridge system, Locking plate, Intramedullary fixation, Clinical outcomes

## Abstract

**Background:**

The aim of this study was to explore the efficacy of a novel intramedullary fixation technique using the ortho-bridge system (OBS) for midshaft clavicle fractures.

**Methods:**

A total of 63 patients were included in this study: 35 underwent plate internal fixation (LP group) and 28 underwent OBS intramedullary fixation (OBS group). Surgical time, intraoperative blood loss, incision length, fracture healing time, removal of the internal fixation agent, visual analog scale (VAS) score for shoulder pain, Constant–Murley shoulder score and complication occurrence were compared between the two groups.

**Results:**

Preoperative general data, such as sex, age and fracture type, were not significantly different between the two groups (*P* > 0.05). However, the OBS group exhibited better outcomes than the LP group exhibited in terms of surgical time, intraoperative blood loss and total incision length (*P* < 0.05). Additionally, the OBS group exhibited a significantly shorter fracture healing time and internal-fixation removal time than the LP group exhibited (*P* < 0.05). The VAS scores on postoperative day 1, week 1, month 1 and month 3 were lower in the OBS group than in the LP group (*P* < 0.05). Furthermore, the Constant–Murley shoulder scores at 1, 3, and 6 months postoperatively were higher in the OBS group than in the LP group (*P* < 0.05), with no significant difference at 1 year after surgery (*P* > 0.05). None of the patients in the OBS group experienced scarring of the surgical incision, and 6 patients in the LP group experienced scarring of the surgical incision. Finally, the complication incidence in the OBS group was lower than that in the LP group.

**Conclusion:**

For midshaft clavicle fractures, OBS intramedullary fixation is better than locking-plate internal fixation because it led to less trauma, a faster recovery, better efficacy, and better esthetic outcomes and comfort. Therefore, this technique may have potential as a novel treatment for midshaft clavicle fractures.

*Level of evidence*: III, retrospective observational study.

## Introduction

Clavicle fractures are common acute fractures that account for 4–10% of adult fractures. Among these fractures, midshaft fractures (Robinson type II) constitute approximately 80% of all clavicle fractures. Furthermore, a midshaft clavicle fracture is more likely than a lateral clavicle fracture to be displaced due to muscle pulling [1]. These fractures are commonly caused by traffic accidents and falls during activities and are often accompanied by injuries such as rib fractures, spinal fractures and pneumothorax [2, 3]. Nonsurgical treatments for midshaft fractures can cause deformity during the healing process and commonly result in nonunion, which leads to shoulder deformity and chronic pain. Moreover, active surgical intervention is recommended for displaced or unstable clavicle fractures [4, 5]. Surgical approaches mainly include intramedullary nail fixation, plate and screw fixation, and ortho-bridge system (OBS) extramedullary fixation [4, 6, 7, 10]. However, all these procedures have corresponding limitations, and there is no gold-standard treatment protocol currently available [6, 7]. Plate fixation has disadvantages, such as significant tissue trauma, a compromised blood supply to the fracture site, and an increased risk of injury to the supraclavicular nerve. Compared to intramedullary fixation, this technique carries a relatively high risk of postoperative complications such as tissue damage, local skin agitation and incisional scarring hyperplasia [8–10]. Locking-plate osteosynthesis provides strong internal fixation during primary fracture healing and is prone to stress shielding, which leads to complications such as delayed bone healing and nonhealing [8, 9, 11]. In contrast, intramedullary nail osteosynthesis involves elastic fixation with micromotor stress at the fracture end, which leads to secondary fracture healing. This conforms to the current BO (biological osteosynthesis) principle of fracture fixation; that is, the biological fixation mode of indirect reduction, elastic fixation and indirect healing [12, 13]. Although clinical studies on intramedullary fixation for clavicle fractures are becoming increasingly common, complications, including unreliable intramedullary device fixation, loosening, displacement and soft-tissue irritation due to protrusion of the nail end, have been highlighted [12, 14, 15].

Therefore, in this study, a novel intramedullary fixation technique using OBS is proposed for midshaft clavicle fractures. The technique involves first inserting the connecting rod from the distal clavicle (acromion extremity) into the medullary cavity of the clavicle to the proximal sternoclavicular clavicle joint and then fixing the end of the rod by installing 1–2 screws in the distal clavicle. If the length of the distal clavicle was sufficient, two screws were selected for fixation. To our knowledge, no other studies have reported this technique. Therefore, the aim of this study was to compare the clinical efficacy of OBS intramedullary fixation with that of locking plate fixation for treating displaced or shortened midshaft clavicle fractures.

## Patients and methods

### Clinical data

A total of 78 patients with mid-clavicle fractures were treated at our hospital from June 2020 to October 2022; 10 opted for conservative treatment and 5 were lost to follow-up. Based on the exclusion and inclusion criteria, a total of 63 patients were included in this retrospective observational study; 35 patients underwent plate internal fixation (LP group) and 28 underwent OBS intramedullary fixation (OBS group). This study was approved by the ethics committee of the Fifth Affiliated Hospital (Zhuhai) of Zunyi Medical University (approval number: [2021] 2021ZH0088). All patients signed informed consent forms permitting the use of their data for further research and publication.

### Eligibility criteria for the study patients

The inclusion criteria for patients were as follows: (1) non-open fractures within 3 weeks of injury; (2) adequate preoperative preparation with reliable data; (3) displaced midshaft clavicle fractures (Robinson type IIA2 or IIB1) [16]; and (4) aged ≥ 18 years. The exclusion criteria for patients were as follows: (1) pathological fractures; (2) infection at the surgical site preoperatively; (3) the presence of other injuries or diseases affecting the function of the affected-side shoulder joint; (4) unable to tolerate surgery due to poor physical condition or severe internal medical diseases; and (5) incomplete follow-up data or were followed up for less than 12 months.

### Surgical methods

A team experienced in trauma surgery performed the surgeries for all patients under a brachial plexus block or general anesthesia. The patients in both groups were placed in the supine position with a pillow under the affected scapula first, and then the surgical area was routinely disinfected and draped.

**Fig. 1:**
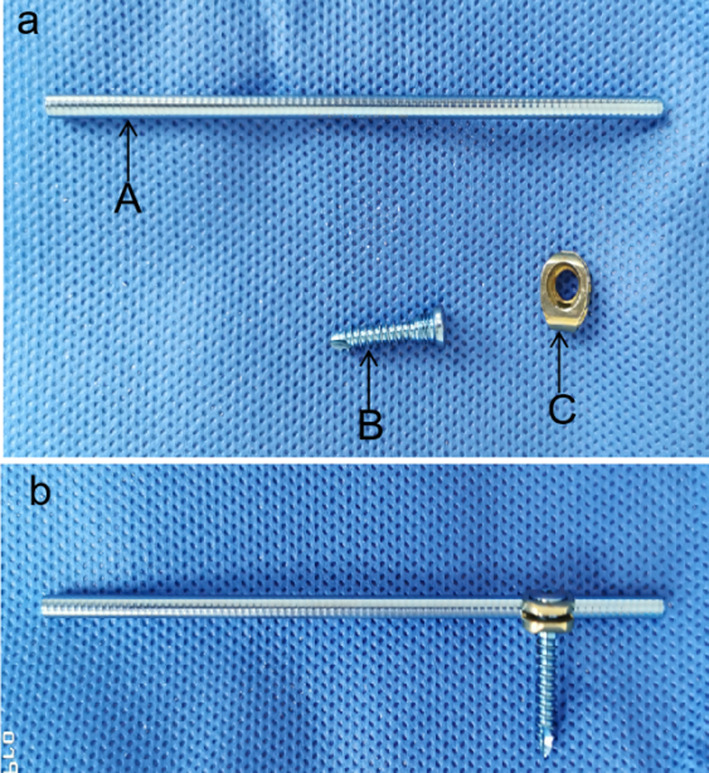
Physical appearance of the ortho-bridge system. **a** Assembly accessories: connecting rod (*A*), locking screw (*B*) and fixation block (*C*). **b** Schematic diagram of the individulized assembly, with one screw, rod and block each

**Fig. 2:**
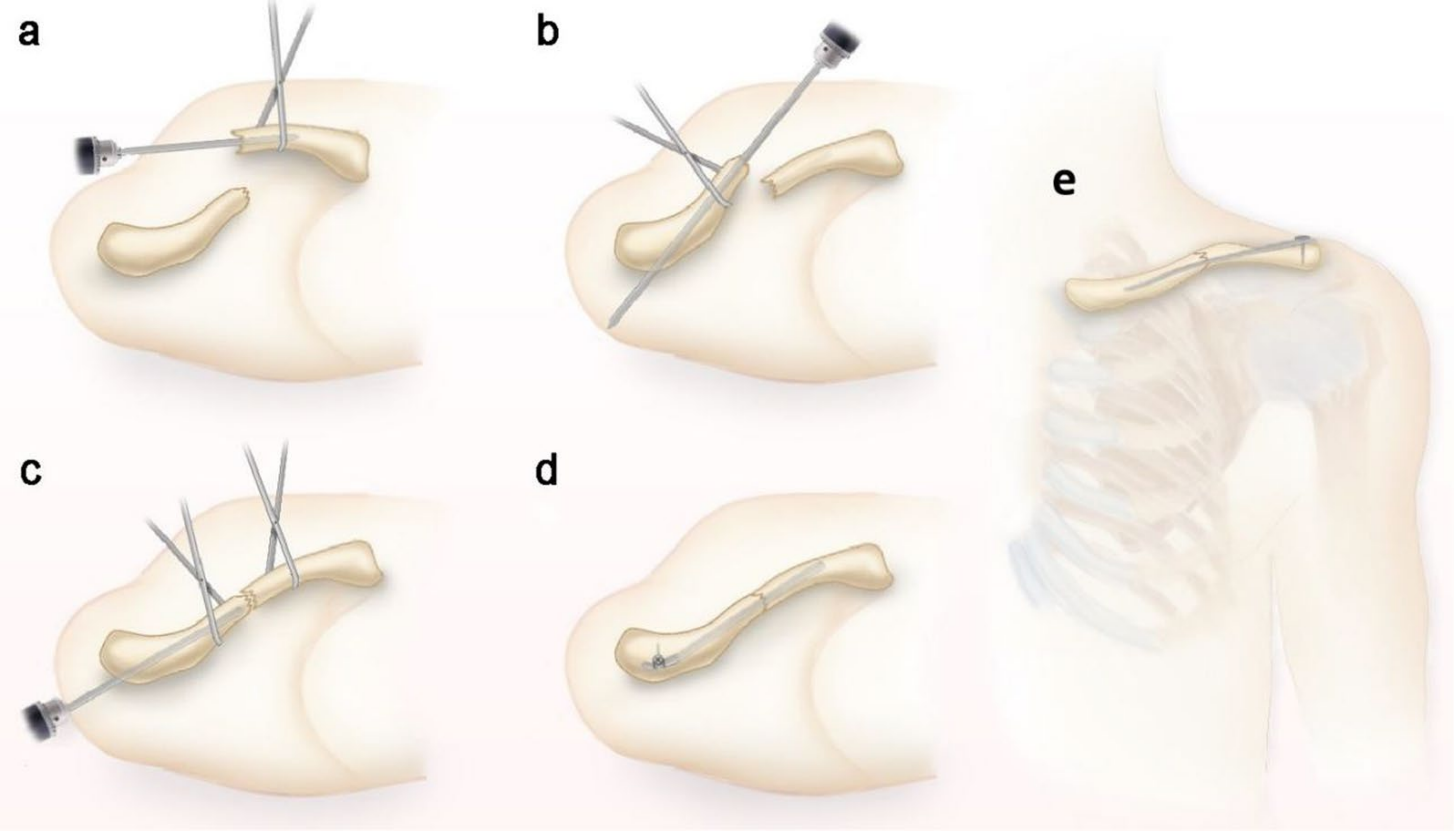
OBS intramedullary fixation technique. **a** The K-wire was drilled into the medial fracture fixed by Kocher forceps. **b** The K-wire was drilled into the lateral fracture end fixed by Kocher forceps. **c** The connecting rod was drilled retrogradely into the medullary cavity of the reduced fracture. **d** The protruding end of the connecting rod was cut off at a suitable length and bent and fixed using the screws. **e** Postoperative orthopedic view of the fracture

Surgical treatment in the OBS group (OBS fixators are detailed in Fig. 1) was as follows. A 2-cm incision was made at the fracture site of the clavicle, followed by layer-by-layer exposure of the fracture end. After the soft-tissue blood clot at the fracture end was cleared, the inner fracture end was gently lifted using a Kocher clamp. A 3-mm-diameter Kirschner wire was inserted retrogradely from the fracture end through the “clavicle medullary cavity” to the medial one-fifth of the clavicle (Fig. 2a). Similarly, the outer fracture end was lifted, expanding anteriorly until the Kirschner wire pierced the cortical bone on the dorsolateral side of the acromial end of the clavicle (Fig. 2b). An incision of approximately 1–2 cm was made at the site where the Kirschner wire pierced the skin to expose the dorsal opening of the distal clavicle, after which the Kirschner wire was removed. Based on the shape of the clavicle, a prebent connecting rod (3 mm in diameter; Weiman Company, Tianjin) was inserted into the medullary cavity of the distal clavicle from the opening at the acromial end (Fig. 2c). Using the connecting rod as a lever, the near end of the fracture was held in place by an assistant (via a Kocher clamp) for reduction under direct vision. The connecting rod was then passed retrogradely through the fractured end to the proximal medullary cavity without piercing the cortical bone at the proximal end. Finally, the tail of the connecting rod outside the acromial end was bent against the cortical bone, and one or two fixation blocks and screws were installed for fixation (Fig. 2d, e). At the fracture end, with obvious displacement of the fracture block, the block was set and fixed in its original position with the aid of suture ligation or tension screws.

Surgical treatment in the LP group was as follows. An approximately 10-cm-long vertical incision was made at the patient's clavicle fracture site. The area was incised layer by layer to expose the area above the clavicle, ensuring that the supraclavicular nerve is protected. The fracture ends were exposed, and the incarcerated soft tissue was cleaned. The fracture was reduced under direct visualization, and temporary fixation was achieved using two 1.5-mm Kirschner wires. An “S” locking plate (Best Biotechnical Company, Beijing) of a suitable length was placed above the clavicle, and screws of an appropriate size were inserted at both ends of the locking plate after drilling and measuring. All patients underwent fluoroscopy to confirm the appropriate placement of the internal-fixation devices and the correct alignment of the fracture fragments before layer-by-layer wound closure.

### Postoperative management

Both groups were subjected to the same postoperative management. Routine postoperative care included methods to reduce swelling, prevent infection and relieve pain. Within 24 h after surgery, the wound dressings were changed, and both patient groups underwent imaging examination. The affected limb was supported with a forearm sling for 3–4 weeks. Patients were encouraged to perform passive motion exercises starting on postoperative day 2. After 2 weeks, all patients were instructed to gradually initiate active shoulder exercises.

### Patient assessment

Postoperative follow-up examinations were conducted and relevant data were collected every 2–4 weeks by two surgeons from our team. The data that were collected were the operation time, intraoperative blood loss, total length of postoperative incisions (the lengths of the two incisions were added together in the OBS group), fracture healing time, and removal time of the internal-fixation devices. Postoperative follow-up examinations were performed at 2- to 4-week intervals to assess the time to fracture healing based on the bone healing criteria, and the last follow-up examination was scheduled. The bone healing criteria for the fracture included the following [17]: (1) no local pressure or percussion pain; (2) no abnormal local activity; and (3) a blurred fracture line with bone trabeculae passing through the fracture line on imaging (X-ray or CT). Additionally, VAS scores were recorded to assess patient pain levels at 1 day, 1 week, and 1, 3, 6 and 12 months after surgery. Shoulder joint function was further assessed by using the Constant–Murley shoulder scale [18] at 1, 3, 6 and 12 months after surgery, where a higher score indicated better shoulder joint function.

The cosmetic effects were observed at each follow-up examination, and complications were recorded. Cosmetic effects: whether there was obvious hypertrophic scarring of the skin incision. Complications: subclavian-nerve and blood-vessel injury, soft-tissue irritation, internal fixation failure (e.g., screw loosening or denailment; a bent or broken plate; loss of reduction), incision infection, skin paresthesia, fracture malunion, delayed union, nonunion, refracture after internal-fixation removal, chronic shoulder pain after surgery, etc.

### Statistical analysis

The statistical analysis was performed using SPSS software (version 29; IBM, USA). Continuous variables were tested for a normal distribution with the Kolmogorov–Smirnov test. Normally distributed quantitative data are expressed as mean ± standard deviation (SD) and were compared using two-sample independent* t* tests. Variables with a nonnormal distribution are expressed as median (interquartile range). The Mann–Whitney* U* test was used for nonnormal distributions. Categorical data are expressed as percentages. Differences between groups were analyzed using chi-square tests or Fisher’s exact test. All the statistical analyses were two-tailed, with the significance level set at* α* = 0.05.

Sample size was calculated with PASS15.0 software. Based on previous studies, we estimated the effect size we hoped to detect and set the power at 80% with an* α* of 0.05 [19]. This analysis suggested that a minimum of 25 subjects per group were needed to have adequate power to test our hypotheses. Post-hoc power analysis was conducted utilizing G*Power 3.1.9.4 software (Universität Kiel, Germany) to determine whether the observed sample size provided sufficient power to detect statistically significant differences between groups. A two-tailed* t* test was used to compare the mean values of the two independent groups of participants in the LP group and the OBS group. Based on the sample sizes of 28 patients in the OBS group and 35 patients in the LP group, a medium effect size of 0.5, and an* α* of 0.05, the post-hoc analysis indicated that the power for this comparison was 0.874, which was greater than 0.8.

## Results

### Comparison of the basic characteristics between the two groups

A total of 63 patients with midshaft clavicle fractures treated in our hospital from June 2020 to October 2022 were included after applying the inclusion and exclusion criteria. There were no significant differences in the basic characteristics between the two groups before surgery (*P* > 0.05), indicating group comparability (Table 1).

**Table 1:** Comparison of the basic characteristics between the two groups before surgery

Variables	OBS group	LP group	Statistical values	*P*
Number of patients (*n*)	28	35		
Sex (male/female)	16/12	24/11	*χ*^2^ = 0.87	0.34
Age (years, $\overline{x}$ ± SD)	38.9 ± 15.80	38.74 ± 13.39	*t* = 0.05	0.96
Fracture side (left/right)	10/18	18/17	*χ*^2^ = 1.55	0.21
Cause of fracture			*χ*^2^ = 4.82	0.19
Car accident	10	15		
Falling from a two-wheeler	11	13		
Falling when walking	1	5		
Other reasons	6	2		
Robinson type			*χ*^2^ = 2.36	0.12
IIA2	15	12		
IIBl	13	23		
Combined injuries			*χ*^2^ = 2.69	0.31
Fractured ribs	7	10		
Cranial injury	5	2		
Soft-tissue injury	2	5		
Time from injury to surgery (days, $\overline{x}$ ± SD)	2.34 ± 2.06	2.21 ± 2.38	*t* = 0.22	0.82
Follow-up time (months, $\overline{x}$ ± SD)	14.61 ± 1.32	15.29 ± 2.01	*t* = −1.54	0.13

*P* < 0.05 vs. the LP group

### Comparison of surgical outcomes between the two groups

All the patients were followed up for 12–24 months (14.98 ± 1.76 months). The OBS group exhibited a significantly shorter surgical time (57.96 ± 10.23 min) and less intraoperative blood loss (20.00 ml) as well as a shorter total incision length (4.80 ± 0.74 cm) than the LP group exhibited (72.69 ± 12.54 min, 50.00 ml, and 10.54 ± 1.58 cm; *P* < 0.05). Furthermore, the OBS group had a significantly shorter fracture healing time and earlier removal of the internal fixation agent than the LP group had (*P* < 0.05) (Table 2).

**Table 2:** Comparison of surgery outcomes between the two groups

	Number of patients	Operation time (min; $\overline{x}$ ± SD)	Intraoperative blood loss [ml; M (Q1, Q3)]	Total length of incision (cm;$\overline{x}$ ± SD)	Fracture healing time (weeks;$\overline{x}$ ± SD)	Removal time of internal fixation (months;$\overline{x}$ ± SD)
OBS group	28	57.96 ± 10.23	20.00 (10.00, 28.75)	4.80 ± 0.74	11.32 ± 1.56	7.50 ± 1.16
LP group	35	72.69 ± 12.54	50.00 (20.00, 50.00)	10.54 ± 1.58	15.13 ± 1.26	13.42 ± 2.12
*t*/*z*		−5.015	−4.373	−19.071	−10.693	−13.281
*P*		0.000	0.000	0.000	0.000	0.000

*P* < 0.05 vs. the LP group

### Comparison of pain severity between the two patient groups at different time points

The pain VAS scores of the OBS group were significantly lower than those of the LP group at 1 day, 1 week, 1 month and 3 months after surgery (*P* < 0.05), but there were no significant differences in the pain VAS scores at 6 months and 1 year after surgery between the two groups (*P* > 0.05) (Table 3).

**Table 3:** Comparison of postoperative VAS scores for pain between the two groups (scores, $\overline{x}$ ± SD)

Time point	Postoperative VAS score for pain		*P*
	OBS group	LP group	
1 day after surgery	4.71 ± 0.75	5.85 ± 0.68	0.00
1 week after surgery	3.48 ± 0.63	4.27 ± 0.71	0.00
1 month after surgery	2.13 ± 0.69	2.77 ± 0.55	0.00
3 months after surgery	1.50 ± 0.64	2.11 ± 0.77	0.00
6 months after surgery	0.68 ± 0.48	0.77 ± 0.60	0.48
12 months after surgery	0.28 ± 0.44	0.43 ± 0.48	0.18

*P* < 0.05 vs. the LP group

### Comparison of postoperative Constant shoulder scores between the two groups

The Constant shoulder scores of the OBS group were significantly greater than those of the LP group at 1 month, 3 months and 6 months after surgery (*P* < 0.05). However, no significant difference was detected between the two groups at 1 year after surgery (*P* > 0.05) (Table 4).

**Table 4:** Comparison of postoperative Constant shoulder scores between the two groups ($\overline{x}$ ± SD)

Time point	Postoperative Constant shoulder score		*P*
	OBS group	LP group	
1 month after surgery	83.21 ± 1.68	71.17 ± 3.11	0.00
3 months after surgery	89.68 ± 2.34	85.66 ± 1.57	0.00
6 months after surgery	94.43 ± 1.53	90.20 ± 2.18	0.00
12 months after surgery	97.71 ± 1.51	97.03 ± 1.62	0.09

*P* < 0.05 vs. the LP group

### Comparison of cosmetic outcomes and complications between the two groups

Cosmetic outcomes: none of the patients in the OBS group had surgical incision scarring, but there were 6 patients in the LP group with surgical incision scarring, and the difference between the two groups was statistically significant (*P* < 0.05).

Complications: no cases of incision infection, nonunion of fractures, or internal fixation failure were reported in either group. All surgical incisions healed primarily. In the OBS group, 1 patient exhibited nail-end protrusion. At 5 months after surgery, the nail end pierced the skin, causing a local chronic inflammatory reaction.

**Fig. 3:**
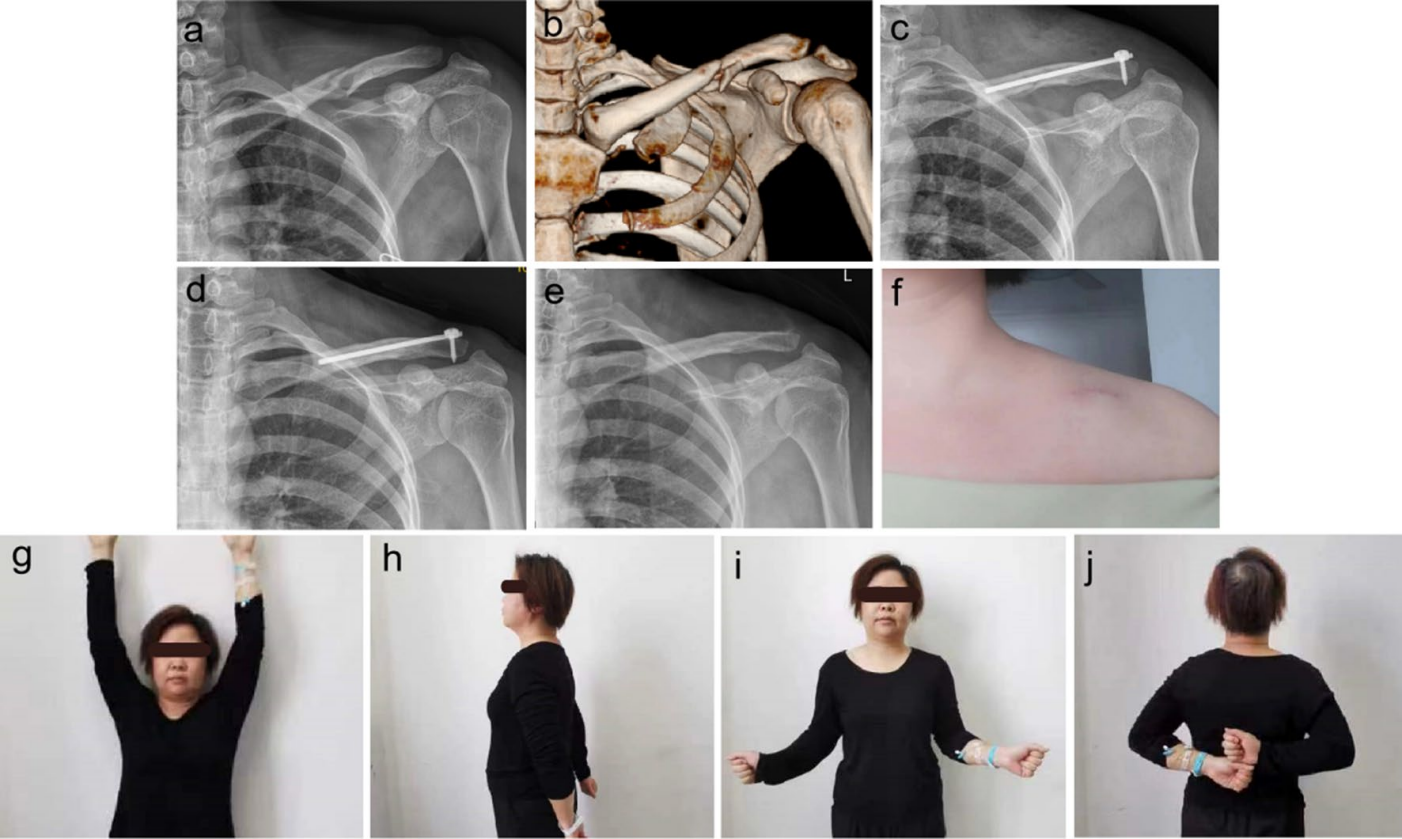
Case of a 34-year-old female who experienced a traffic accident. **a**, **b** The preoperative orthoptic radiograph and CT 3D reconstruction of the left clavicle showed a mid-clavicle fracture. **c** At 2 d postoperatively, an orthoptic radiograph of the left clavicle showed a well-replaced fracture. **d** At 10 d postoperatively, the skin at the incision was esthetically pleasing, with no scars. **e** At 7 months postoperatively, an orthoptic radiograph of the left clavicle showed good fracture healing. **f** At 7 months postoperatively, an orthoptic radiograph of the left clavicle after removal of the internal fixation showed a normal clavicle morphology. **g**–**j** By 7 months postoperatively, the function of the shoulder joint had returned to normal

**Fig. 4:**
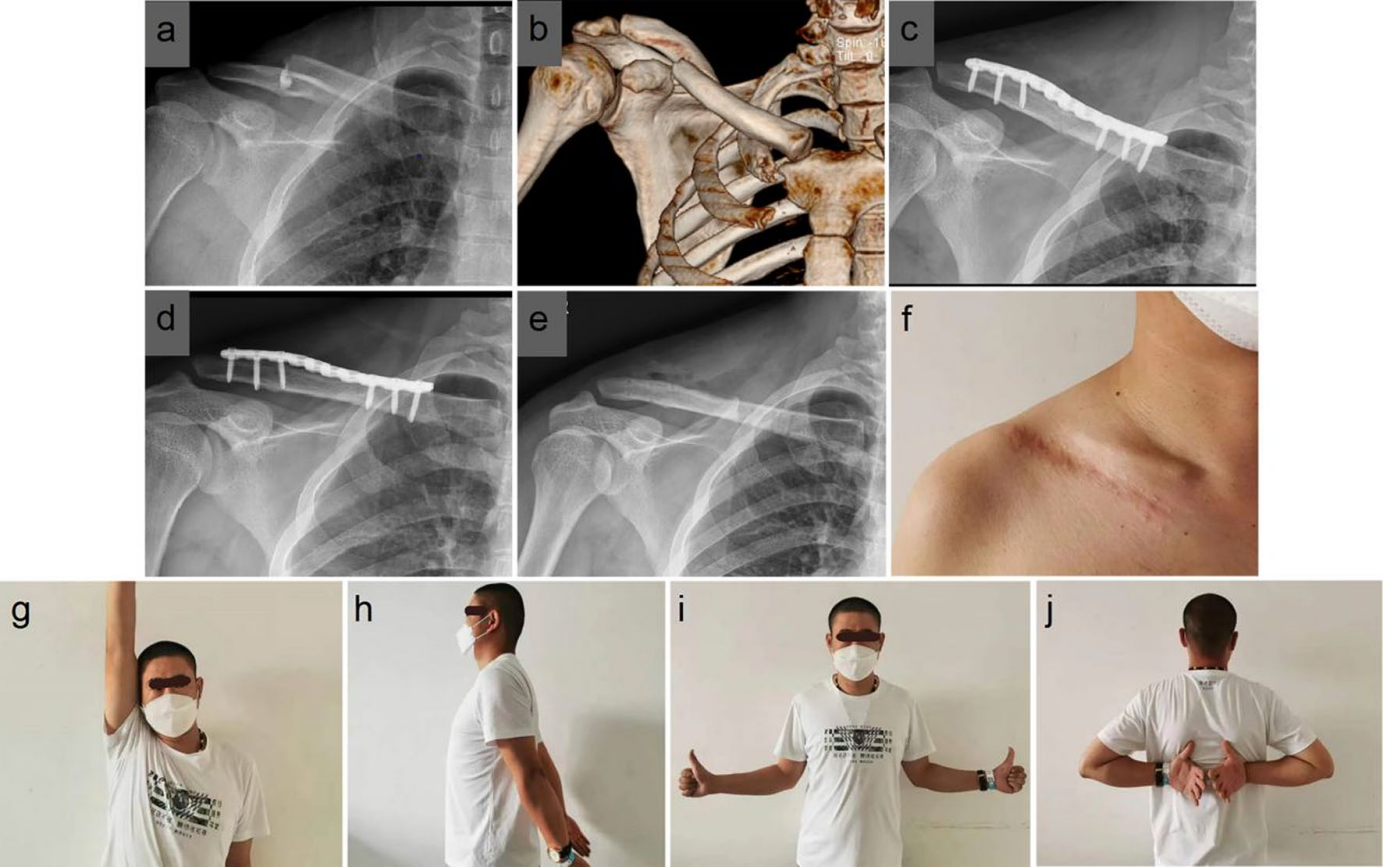
A 36-year-old male who sustained a fracture during a traffic accident. **a**, **b** Preoperative orthogonal X-ray and CT 3D reconstruction of the right shoulder showed a fracture of the middle clavicle. **c** Orthogonal X-ray of the right shoulder 2 d after surgery showed good fracture repositioning. **d** Orthogonal X-ray of the right shoulder 1 year after surgery showed good fracture healing. **e** Plain radiograph of the right shoulder 1 year after removal of the locking plate showed a basically normal clavicle morphology with bone loss at the nail path. **f** Skin scarring of the incision 1 year after surgery. **g**–**j** The patient's shoulder function had returned to normal at the 1-year-postoperative review

In the LP group, 3 patients exhibited obvious subcutaneous protrusion of the internal-fixation device, causing soft-tissue irritation. Additionally, 2 patients presented with localized abnormal skin sensation; 1 suffered refracture 1 week after the removal of the plate and underwent internal fixation of the plate and bone grafting again. The locking plate was removed when the bone healed 1.5 years after surgery. Moreover, the incidence of complications in the OBS group [(3.57% (1/28)] was significantly lower than that in the LP group [17.10% (6/35)]. Representative cases are illustrated in Figs. 3 and 4.

## Discussion

In this retrospective study, we compared the clinical outcomes of OBS intramedullary fixation with those of plating for treating midshaft clavicle fractures. We revealed that surgical time, intraoperative blood loss and total incision length were significantly greater in the OBS group than in the control group, illustrating that this new technique is convenient and causes minimal trauma. Additionally, there was less pain early after surgery, earlier shoulder joint functional recovery, a shorter fracture healing time and fewer complications in the OBS group than in the LP group, showing that patients benefited more from the less invasive procedure of the OBS intramedullary fixation technique.

In recent years, intramedullary fixation has emerged as a minimally invasive and clinically effective surgical treatment option for midshaft clavicle fractures [12, 14, 20, 21]. However, the double-arch shape and narrow, irregular medullary cavity of the clavicle have hindered the clinical application of intramedullary fixation devices [22, 23]. Furthermore, Harmouchi et al. [24] reported that the Kirschner wires used for fixing intramedullary clavicle fractures were highly prone to displacement; they migrated to critical areas, such as the heart or lungs, causing fatal injuries. To reduce the risks of surgical trauma and postoperative complications, numerous researchers have explored measures to improve this technique [12, 14, 25]. However, the intramedullary fixation devices most commonly used in clinical practice are also prone to problems such as nail loosening, soft-tissue irritation and fixation failure [15]. Moreover, unstable intramedullary fixation greatly limits the treatment of comminuted fractures [12, 14, 15]. Many studies have reported the use of OBS for the extramedullary fixation of clavicle fractures, which potentially facilitates indirect fracture reduction without stripping the periosteum and promotes fracture healing via three-dimensional elastic fixation [26, 27]. However, this approach has some limitations, including increased risks of hematoma, acromioclavicular pain, implant-related pain, and subcutaneous prominence of the nail rod [7]. The novel intramedullary fixation technique described in our study, OBS intramedullary fixation for midshaft clavicle fractures, addresses these problems.

The OBS intramedullary fixation technique has several advantages that make it a promising surgical approach for treating midshaft clavicle fractures. First, this new technique is convenient and causes minimal trauma. The following are advantages. (1) The operation is easy to perform in hospitals due to the small size of the incision and simple operation. (2) The diameter of the connecting rod is only 3 mm, and the rod is flexible and malleable, allowing the rod tail to be easily bent and shaped, while the screws are flexible enough to be inserted from multiple directions. )3) After the fracture has completely healed, a small incision is made at the end of the nail rod for removal. Our study revealed that, compared to plate fixation, OBS intramedullary fixation required smaller surgical incisions and a shorter operation and led to less intraoperative blood loss. At the 1-year follow-up examination, there was no significant difference in the Constant–Murley shoulder score or VAS score between the two groups. Additionally, the OBS group exhibited no scars or numbness at the incision site, indicating improved skin aesthetics and comfort.

Second, the OBS intramedullary fixation technique involves minimally invasive incisions on the lateral and midportion of the clavicle, resulting in minimal surgical trauma. During this procedure, the supraclavicular nerve was avoided. In contrast, steel-plate fixation requires longitudinal incisions along the clavicle, potentially damaging the supraclavicular nerve and causing more surgical trauma [28]. Given the impact of incision choice on the patient’s postoperative skin sensation, recent studies have explored alternative incision placements. Ankers et al. [29] performed a retrospective review and showed that compared to conventional transverse incisions, an oblique incision along Langer’s lines did not reduce the rate of complications following the fixation of displaced middle-third clavicle fractures. Longitudinal vertical incisions or oblique incisions following the nerve course are likely to reduce the risk of injury to iatrogenic supraclavicular nerves [30, 31]. Whether the nerves are protected when these methods are performed depends on the surgeon’s experience, and the implementation of nerve protection strategies is time-consuming [31]. These findings showed that although neither group had significant differences in pain at 6 and 12 months after surgery, the OBS group demonstrated significantly lower VAS pain scores than the LP group at 1 day, 1 week, 1 month and 3 months after surgery. Thus, OBS intramedullary fixation may reduce early and midterm postoperative pain in patients. Moreover, in the present study, the total length of the incision in the OBS group (4.80 ± 0.74 cm) was significantly shorter than that in the LP group (10.54 ± 1.58 cm). None of the patients in the OBS group had surgical incision scarring, whereas 6 patients in the LP group had surgical incision scarring, which indicates that OBS intramedullary fixation has a better cosmetic effect than steel-plate internal fixation. The use of OBS intramedullary fixation for the treatment of midclavicular fractures provides significant pain relief in the early to mid-postoperative period as well as good cosmetic outcomes.

Third, related studies have shown that intramedullary fixation is suitable for preserving the biomechanical characteristics of the normal clavicle, is minimally invasive and does not strip the periosteum, and provides dynamic elastic fixation. Additionally, the healing time of clavicle midshaft fractures and the recovery time of shoulder joints have been reported to be significantly shorter with intramedullary fixation than with plate fixation [32, 33]. In line with these results, our study revealed that the OBS group had a shorter fracture healing time (11.32 ± 1.56 weeks) than the LP group did (15.13 ± 1.26 weeks). Furthermore, the Constant–Murley shoulder scores at 1 month, 3 months and 6 months after surgery were greater in the OBS group (83.21 ± 1.68, 89.68 ± 2.34, and 94.43 ± 1.53, respectively) than in the LP group (71.17 ± 3.11, 85.66 ± 1.57, and 90.20 ± 2.18, respectively), indicating that shoulder joint function was better and functional recovery occurred earlier in the OBS group than in the LP group.

Finally, many studies [8–10] have revealed that patients with midshaft clavicle fractures who undergo fixation with steel plates are prone to complications such as a noticeable sensation of subcutaneous protrusion of the plate and hypertrophic scar formation at the incision site due to the subcutaneous location of the clavicle. Moreover, the occurrence rate of nonunion or refracture after plate removal has been consistently underestimated [34]. A retrospective study by Zhu et al. [34] revealed that the refracture incidence rate after plate removal was 6.5% (23/352) in patients with midshaft clavicle fractures fixed with plates, emphasizing the likelihood of this complication in high-risk patients, including those with severe fracture comminution, postmenopausal women, and male smokers. Furthermore, recent biomechanical studies have highlighted the issue of insufficient fixation strength of intramedullary devices for clavicle fractures, while clinical observations have frequently reported problems such as loosening and displacement of intramedullary nails, the protrusion of nail ends that irritate the skin and soft tissue, and the failure of internal fixation [12, 14, 15]. In this study, the observed complication rate in the OBS group was significantly lower than that in the LP group [3.57% (1/28) vs. 17.10% (6/35)] and also lower than previously reported complication incidence rates associated with intramedullary fixation of clavicle fractures [34–36]. In the OBS group in our study, 1 patient presented with subcutaneous protrusion of the rod end postoperatively. At 5 months after surgery, this complication resulted in chronic inflammation of the surrounding soft tissue due to subsequent skin damage and exposure of the rod end. This issue may be attributed to the excessive length or inadequate bending of the rod end during surgery, which was further compounded by the patient’s lean physique and limited subcutaneous soft tissue. This complication should be considered and avoided as much as possible in clinical practice.

Under physiological conditions, the clavicle is a non-weight-bearing bone that does not require a strong internal fixation device to maintain a fracture reduction [31, 34]. After fixation, only the tension from the opposing muscles is sufficient to maintain the original alignment of the fracture [32, 35, 37]. The OBS intramedullary fixation technique in this study involved elastic fixation, which is in line with the current “BO” concept of fracture fixation and the cosmetic outcomes desired by the public. In contrast, plate fixation, an eccentric fixation approach, changes the original mechanical transmission mechanism of the clavicle, leading to significant stress shielding [38]. Moreover, the high stiffness of the plate and the sustained stress shielding effect during the later stages of fracture healing affect callus remodeling and might cause delayed fracture healing and reduce bone hardness after healing, potentially resulting in refracture after plate removal [39, 40].

In conclusion, the results of this study showed that the OBS intramedullary fixation technique for midclavicular fractures is not only clinically effective but also advantageous in that it is a simple operation, minimally invasive, and provides good cosmetic outcomes, as it is a method of elastic fixation. This technique is recommended, but there are several limitations of this study. First, this study was retrospective in nature. Nevertheless, the preoperative inclusion criteria were consistent between the two groups. Additionally, all surgeries were performed by the same surgeons, and postoperative management and follow-up examinations were performed using the same approach. Second, the VAS score for pain was limited by subjective factors, and individual differences among the patients might have caused the VAS scores to be biased. Last, the sample size of this study was small. Hence, multicenter large-sample prospective studies are needed to obtain more accurate results.

## Conclusion

In this study, we revealed that OBS intramedullary fixation is better than locking-plate internal fixation of midshaft clavicle fractures in terms of convenience and because it leads to less early postoperative pain, a shorter fracture healing time, better cosmetic outcomes, greater comfort during incision, and fewer complications. Therefore, OBS intramedullary fixation may be a new clinical option for treating midshaft clavicle fractures.

## Data Availability

Data sharing is not applicable to this article as no datasets were generated or analyzed during the current study.

## Abbreviations

OBS: Ortho-bridge system
VAS: Visual analog scale
BO: Biological osteosynthesis
